# Statistics-based model for prediction of chemical biosynthesis yield from *Saccharomyces cerevisiae*

**DOI:** 10.1186/1475-2859-10-45

**Published:** 2011-06-21

**Authors:** Arul M Varman, Yi Xiao, Effendi Leonard, Yinjie J Tang

**Affiliations:** 1 Department of Energy, Environmental and Chemical Engineering, Washington University, St. Louis, MO 63130, USA; 2 Department of Chemical Engineering, Massachusetts Institute of Technology, Cambridge, MA 02139, USA

## Abstract

**Background:**

The robustness of *Saccharomyces cerevisiae *in facilitating industrial-scale production of ethanol extends its utilization as a platform to synthesize other metabolites. Metabolic engineering strategies, typically via pathway overexpression and deletion, continue to play a key role for optimizing the conversion efficiency of substrates into the desired products. However, chemical production titer or yield remains difficult to predict based on reaction stoichiometry and mass balance. We sampled a large space of data of chemical production from *S. cerevisiae*, and developed a statistics-based model to calculate production yield using input variables that represent the number of enzymatic steps in the key biosynthetic pathway of interest, metabolic modifications, cultivation modes, nutrition and oxygen availability.

**Results:**

Based on the production data of about 40 chemicals produced from *S. cerevisiae*, metabolic engineering methods, nutrient supplementation, and fermentation conditions described therein, we generated mathematical models with numerical and categorical variables to predict production yield. Statistically, the models showed that: 1. Chemical production from central metabolic precursors decreased exponentially with increasing number of enzymatic steps for biosynthesis (>30% loss of yield per enzymatic step, P-value = 0); 2. Categorical variables of gene overexpression and knockout improved product yield by 2~4 folds (P-value < 0.1); 3. Addition of notable amount of intermediate precursors or nutrients improved product yield by over five folds (P-value < 0.05); 4. Performing the cultivation in a well-controlled bioreactor enhanced the yield of product by three folds (P-value < 0.05); 5. Contribution of oxygen to product yield was not statistically significant. Yield calculations for various chemicals using the linear model were in fairly good agreement with the experimental values. The model generally underestimated the ethanol production as compared to other chemicals, which supported the notion that the metabolism of *Saccharomyces cerevisiae *has historically evolved for robust alcohol fermentation.

**Conclusions:**

We generated simple mathematical models for first-order approximation of chemical production yield from *S. cerevisiae*. These linear models provide empirical insights to the effects of strain engineering and cultivation conditions toward biosynthetic efficiency. These models may not only provide guidelines for metabolic engineers to synthesize desired products, but also be useful to compare the biosynthesis performance among different research papers.

## Background

**Figure 1:**
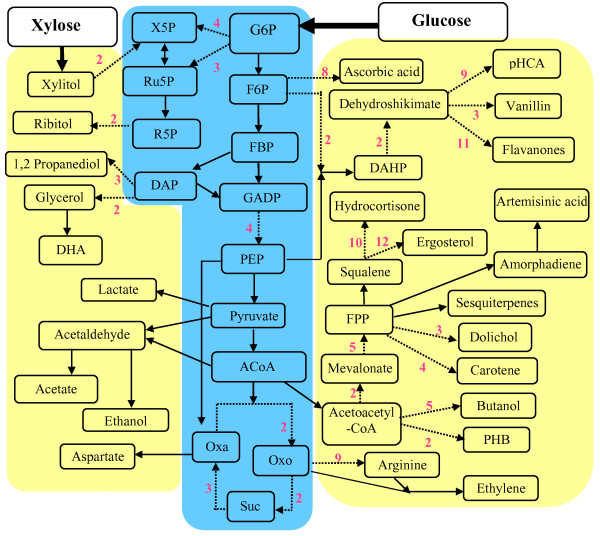
**Metabolic pathways for the biosynthesis of major products**. The blue box represents central metabolism and the yellow box represents secondary metabolism. Solid arrows signify single step reaction and dotted arrows (→) signify multiple steps. Abbreviations**: **ACoA - Acetyl-CoA; DAP - Dihydroxyacetone-Phosphate; DAHP - 3-Deoxy-D-Arabino-Heptulosonate-7-Phosphate; DHA - Dihydroxyacetone; F6P - Fructose-6-Phosphate; FBP - Fructose 1,6-bisphosphate; G6P - Glucose-6-Phosphate; GADP - Glyceraldehyde-3-Phosphate; Oxa - Oxaloacetate; Oxo - 2-Oxoglutarate; PEP - Phosphoenolpyruvate; PHB - Poly[(R)-3-hydroxybutyrate]; pHCA - p-Hydroxycinnamic acid; R5P - Ribose-5-Phosphate; Ru5P - Ribulose-5-Phosphate; Suc - Succinate; X5P - Xylulose-5-Phosphate.

Producing small-molecule chemicals from microbial biocatalysts offers several advantages. Unlike conventional chemical synthesis which are heavily dependent on petroleum-derived substrates, microbes are able to use renewable materials to synthesize many commodity chemicals and fuels [1] (Figure 1). Due to its scalability, microorganisms are also suitable platforms to synthesize pharmaceutical molecules that are conventionally produced from extracting large amounts of natural resources. Among many industrial microorganisms, the baker's yeast, i.e., *S. cerevisiae *continues to emerge as a preferred production platform [2]. *S. cerevisiae *is typically known for its robustness in fermenting sugars into alcohol. In the recent past, it has also gained importance as a heterologous platform to synthesize many precursors of commodity chemicals and pharmaceuticals [1]. In general, chemical production using whole-cell biocatalysts are achieved by genetic engineering to extend the substrate range of an existing biosynthetic pathway or to introduce new biosynthetic pathways (either derived from other organisms, or completely novel). Rational metabolic engineering approaches then analyze the cellular metabolism and improve production titer by overexpressing rate-limiting enzymes or deleting competing pathways. In general, the actual yield of chemical production is not easily predicted due to the complexity of biological systems and dependency of cultivation conditions. Biological complexities not only include intrinsic properties (such as enzyme kinetics and substrate specificity), but also include enzyme compartmentalization, intracellular signaling, and metabolite transport between eukaryotic cell organelles. Therefore, strain engineering requires multiple rounds of trial-and-error experiments to perform the optimum combination of genetic manipulations. In the present work, we sought to develop mathematical models that could provide *a priori *estimation of chemical production yield from engineered *S. cerevisiae *when given a set of parameters, namely the number of steps in the biosynthetic pathway of interest, genetic modifications, cultivation conditions, and nutrient and oxygen availability. The coefficients of these parameters were obtained from the regression of the yields and production conditions reported by recent literatures. Such model predicted the empirical yields that were lower than the theoretical productivities under "ideal" conditions. The model results could give metabolic engineers guidelines for increasing desired products and for reducing futile attempts.

### Model development

The model defined several important parameters that influenced the efficiency of chemical production from microbial hosts. The first group of parameters accounted for the number of enzymatic steps in the biosynthetic pathway of interest since it had been shown that this parameter was often inversely correlated with microbial product yield [3]. To enumerate the number of enzymatic steps, we introduced two numerical variables in our model, i.e. PRI and SEC. The variable PRI specified the number of enzymatic steps in primary metabolism (Figure 1), e.g. glycolysis that is required to convert sugar (glucose or galactose) to pyruvate. The variable SEC specified the number of enzymatic steps in the subsequent pathway (typically belongs to secondary metabolism), which catalyzed the conversion of central carbon intermediate into the final product of interest. The next group of variables was to capture the effects of genetic modification. Various genetic strategies have been used to implement metabolic engineering [4,5]. For example, promoters with different strength influence production level. However, for the sake of simplifying our model, variations of genetic components used in metabolic engineering strategies were lumped into two ordinal variables, i.e. OVE, and KNO. OVE signified the introduction of multiple copies of genes of native or heterologous origin for the purpose of improving production level. KNO signifies the alteration of branch pathways that might compete with the pathway of interest [6,7]. We further sub-categorized OVE based on the number of modified genes into OVE_C1 _(without "pushing" pathway flux), OVE_C2 _(enhancing 1~2 enzyme activities), and OVE_C3 _(improving a number of key enzyme functions). KNO was also categorized by KNO_C1 _and KNO_C2 _(i.e., without knockout or with knockout, respectively). Table 1 explained the specifications for each sub-category.

**Table 1:** Ordinal variables used in the linear regression model

Ordinal variables	Category 1 (subscript C1)	Category 2 (subscript C2)	Category 3 (subscript C3)
OVE: number of modified genes or pathways	No modified genes or pathways were present.	One or two modified genes or pathways were present.	More than two modified genes or pathways were present.

KNO: number of gene knockouts in known competitive pathways	No gene knockouts were performed.	Gene knockouts were performed.	

NUT: nutrient source	Fermentation occurred in defined medium (only including trace amounts of amino acids or vitamins)	Fermentation occured in a very rich medium.	

INT: Intermediate	Intermediate was not added	Intermediate was added	

CUL: cultivation mode	Fermentation occurred in a shaking flask.	Fermentation occurred in a batch, fed-batch, or continuous feed bioreactor.	

OXY: oxygen conditions	Fermentation occurred in aerobic conditions.	Fermentation occurred under oxygen-limited conditions (anaerobic or micro-aerobic).	

Note: the input of ordinal variables was specified using a binary system, 1 and 0. When a category (e.g., overexpression Category 2) was applied, the value 1 was assigned to OVEc2. Otherwise, the value 0 was assigned.

The yield of metabolite production is also a function of cultivation conditions and nutrient availability. For instance, production of metabolites from a bioreactor is often higher than a shaking flask, due to the increased efficiency of mass transfer of oxygen, substrates, and nutrients. Moreover, culture acidification that often generates cytotoxicity and maintenance burden to the microbial hosts can be mitigated in a bioreactor by automated pH control. Based on these basic properties, we introduced the variable CUL to represent the general property of a cultivation condition. We also introduced the variable OXY and NUT to capture the effects of oxygen availability and nutrient supplementation, respectively [8-10]. Moreover, the variable INT captured the effect of addition of a secondary carbon source which served as a precursor or an intermediate metabolite of the pathway of interest.

Several assumptions were made to simplify our model development. A) Yield calculation was based on the conversion of major carbon substrate to final product if multiple nutrient sources were supplemented (e.g., yeast extract was not treated as the carbon source). B) We calculated the yields based on two factors: initially added carbon substrate in the culture and final measured product. We neglected the unused carbon substrate that remained in the end of the production. C) To calculate enzymatic steps from the carbon source, the model only considered the key route from the major substrate (mostly glucose) to the final products (enzyme steps for co-factors or ATPs synthesis were neglected). D) For product synthesis promoted by the addition of an intermediate, we had no means of differentiating the carbons derived from added precursor or from the carbon substrate (i.e., glucose). To account for the contribution from both carbon sources, the yield calculation was assumed to be an arithmetic mean of the two yields (One yield was based on substrate, e.g., glucose, and the other yield was estimated from the intermediates). Meanwhile, the number of primary steps or secondary steps were also assumed as an arithmetic mean of two data sets (one variable was counted from substrate; the other variable was counted from the intermediate).

Biochemical systems theory [5] states that reaction rates (v_i_) can be described by a general power law expression of the type:(1)

Where X_j _represents the system variables and the parameters α_i_, g_ij _are the constants. Equation (1) yields a linear form in logarithmic coordinates. Based on similar assumptions, our model for yield prediction used system variables (i.e., numerical or categorical variables related to yeast biosynthesis) to describe the relative carbon flux to the final products.(2)

In Equation 2, log_10 _Y was the dependent variable which represented production yield (mol C in product/mol C in primary substrate), given each independent variables β*_i _*[11]. We defined β_0 _as the intercept in Equation 2, which represented the combined contribution of Category 1 of all ordinal variables. β_0 _was defined as:(3)

The ordinal variables (using a binary system) were assigned a value of one if and only if the condition fitted the category in Table 1. Otherwise, the ordinal variables were assigned a value of 0 [12]. (2) To acquire the coefficients in Equation 2 and 3, we compiled data from ~40 publications which described the production of chemicals by *S. cerevisiae *under various experimental conditions. Table 2 summarized the categories assigned to these experimental conditions and the yield of product from our best judgment. Using these data, we performed regression analysis to fit the model via the software package R [13] to find the regression coefficients and P-values. For this study, a variable was statistically significant (90%) if its P-value was below 0.1.

**Table 2:** Dataset used for the linear regression

Reference	Product	Yield	Primary Step	Second Step	OVE_C2	OVE_C3	KNO_C2	NUT_C2	INT_C2	CUL_C2	OXY_C2
[41]	(E, E, E)-Geranylgeraniol	0.00025	10	10	1	0	0	1	0	0	0
[41]	(E, E, E)-Geranylgeraniol	0.014	10	10	0	1	0	1	0	0	0
[41]	(E, E, E)-Geranylgeraniol	0.047	10	10	0	1	0	1	0	0	0
[41]	(E, E, E)-Geranylgeraniol	0.018	10	10	0	1	0	1	0	0	0
[41]	(E, E, E)-Geranylgeraniol	0.031	10	10	0	1	0	1	0	0	0
[41]	(E, E, E)-Geranylgeraniol	0.058	10	10	0	1	0	1	0	0	0
[41]	(E, E, E)-Geranylgeraniol	0.14	10	10	0	1	0	1	0	1	0
[42]	1,2-Propanediol	0.014	4	3	1	0	0	1	0	0	0
[43]	1,2-Propanediol	0.010	4	3	1	0	0	1	0	1	0
[43]	1,2-Propanediol	0.026	4	3	1	0	0	1	0	1	0
[44]	5-epi-aristolochene	0.010	10	9	1	0	1	1	0	0	0
[44]	5-epi-aristolochene	0.0090	10	9	1	0	1	1	0	0	0
[45]	Acetate	0.13	9	2	0	0	1	0	0	1	0
[46]	Acetate	0.015	9	2	0	0	1	0	0	1	0
[47]	Acetate	0.26	9	2	0	1	0	0	0	0	1
[48]	Amorphadiene	0.00049	12	9	1	0	0	0	0	0	0
[48]	Amorphadiene	0.0020	12	9	1	0	0	0	0	0	0
[48]	Amorphadiene	0.0040	12	9	1	0	1	0	0	0	0
[48]	Amorphadiene	0.011	12	9	1	0	1	0	0	0	0
[48]	Amorphadiene	0.016	12	9	0	1	1	0	0	0	0
[48]	Amorphadiene	0.016	12	9	0	1	1	0	0	0	0
[49]	Amorphadiene	0.0080	12	9	1	0	1	0	0	0	0
[49]	Amorphadiene	0.0090	12	9	0	1	1	0	0	0	0
[49]	Amorphadiene	0.011	12	9	0	1	1	0	0	0	0
[49]	Amorphadiene	0.013	12	9	0	1	1	0	0	0	0
[48]	Artemisinic acid	0.0030	12	10	0	1	1	0	0	0	0
[48]	Artemisinic acid	0.011	12	10	0	1	1	0	0	1	0
[50]	Cyanophycin	0.12	10(0)	2(1)	1	0	1	1	1	0	0
[50]	Cyanophycin	0.10	10(0)	2(1)	1	0	1	1	1	0	0
[50]	Cyanophycin	0.15	10(0)	2(1)	1	0	1	1	1	0	0
[51]	Dihydroxyacetone	0.0040	4	3	1	0	0	0	0	0	0
[51]	Dihydroxyacetone	0.034	4	3	1	0	1	0	0	0	0
[52]	D-Lactic acid	0.61	9	1	1	0	1	1	0	0	1
[53]	Dolichol	0.00010	10	11	0	0	0	1	0	0	0
[53]	Dolichol	0.00018	10	11	1	0	0	0	0	0	0
[53]	Ergosterol	0.00015	10	21	0	0	0	1	0	0	0
[53]	Ergosterol	0.00020	10	21	1	0	0	0	0	0	0
[20]	Ethanol	0.55	9	2	1	0	0	0	0	1	1
[20]	Ethanol	0.47	8	2	0	1	0	0	0	1	1
[54]	Ethanol	0.080	8	2	0	1	0	0	0	1	0
[54]	Ethanol	0.12	8	2	0	1	0	0	0	1	1
[54]	Ethanol	0.15	8	2	0	1	0	0	0	1	1
[55]	Ethanol	0.53	9	2	1	0	0	1	0	0	0
[55]	Ethanol	0.20	9	2	1	0	0	1	0	0	0
[55]	Ethanol	0.47	9	2	1	0	0	1	0	0	0
[55]	Ethanol	0.42	9	2	0	0	0	1	0	0	0
[55]	Ethanol	0.36	9	2	0	0	0	1	0	0	0
[46]	Ethanol	0.44	9	2	0	0	1	0	0	1	0
[46]	Ethanol	0.32	8	2	0	0	1	0	0	1	1
[56]	Ethanol	0.52	9	2	1	0	0	0	0	0	0
[47]	Ethanol	0.55	9	2	0	1	0	0	0	0	1
[47]	Ethanol	0.39	9	2	0	1	0	0	0	0	1
[47]	Ethanol	0.51	9	2	0	1	1	0	0	0	1
[57]	Ethylene*	0.00069	13	10	1	0	0	0	0	1	0
[44]	Farnesol	0.036	10	9	0	0	1	1	0	0	0
[58]	Flavanones	0.030	10(0)	14(3)	0	1	0	0	1	0	0
[58]	Flavanones	0.053	10(0)	14(3)	0	1	0	0	1	0	0
[59]	Formate	0.00024	6	7	0	0	1	0	0	0	0
[59]	Formate	0.00030	6	7	0	0	1	0	0	0	0
[60]	Geraniol	0.00011	10	8	1	0	0	0	0	0	0
[60]	Geraniol	0.00019	10	8	1	0	1	0	0	0	0
[60]	Geraniol	0.00019	10	8	1	0	1	0	0	0	0
[61]	Glycerol	0.12	4	2	1	0	0	0	0	1	0
[61]	Glycerol	0.12	4	2	1	0	0	0	0	1	0
[45]	Glycerol	0.41	4	2	1	0	1	0	0	1	0
[45]	Glycerol	0.45	4	2	1	0	1	0	0	1	0
[45]	Glycerol	0.45	4	2	1	0	1	0	0	0	0
[62]	Glycerol	0.49	4	2	0	0	1	1	0	1	0
[62]	Glycerol	0.41	4	2	0	0	1	1	0	1	0
[46]	Glycerol	0.050	4	2	0	0	1	0	0	1	0
[46]	Glycerol	0.037	2	4	0	0	1	0	0	1	1
[63]	Glycerol	0.45	4	2	0	0	1	0	0	1	0
[63]	Glycerol	0.54	4	2	1	0	1	0	0	1	0
[30]	Glycerol 3-phosphate	0.0010	4	1	1	0	1	0	0	0	1
[64]	Hydrocortisone	0.0020	10(0)	19(2)	1	0	0	1	1	0	0
[64]	Hydrocortisone	0.0020	10(0)	19(2)	1	0	1	1	1	0	0
[64]	Hydrocortisone	0.021	10(0)	19(2)	1	0	1	1	1	0	0
[64]	Hydrocortisone	0.026	10(0)	19(2)	1	0	1	1	1	0	0
[65]	Lactate	0.44	9	1	1	0	0	1	0	1	0
[66]	Lactate	0.21	9	1	1	0	0	0	0	1	0
[67]	L-Ascorbic acid	0.14	2(0)	8(2)	1	0	0	0	1	0	0
[67]	L-Ascorbic acid	0.066	2(0)	8(2)	1	0	0	0	1	0	0
[60]	Linalool	8.8 × 10^-5^	10	8	1	0	0	0	0	0	0
[60]	Linalool	2.3 × 10^-5^	10	8	1	0	1	0	0	0	0
[68]	L-Lactic Acid	0.65	9	1	1	0	1	1	0	0	0
[32]	Malate	0.28	11	0	0	1	0	0	0	0	0
[49]	Mevalonate	0.022	12	3	0	1	1	0	0	0	0
[49]	Mevalonate	0.022	12	3	0	1	1	0	0	0	0
[69]	Naringenin	0.0070	8(0)	15(3)	0	1	0	0	1	0	0
[69]	Naringenin	0.0020	8(0)	15(5)	0	1	0	0	1	0	0
[15]	Naringenin	0.00058	10	14	0	1	0	1	0	0	0
[70]	n-Butanol	0.00020	12	**6**	0	1	0	0	0	0	1
[69]	p-Coumaric Acid	0.033	8(0)	12(2)	0	1	0	0	1	0	0
[71]	p-Hydroxycinnamic acid	0.00020	8	12	1	0	0	0	0	0	0
[71]	p-Hydroxycinnamic acid	0.20	8(0)	12(2)	1	0	0	0	1	0	0
[15]	Pinocembrin	6.6 × 10^-5^	10	14	0	1	0	1	0	0	0
[72]	Poly[(R)-3-hydroxybutyrate]	0.00056	10	3	1	0	0	0	0	0	0
[72]	Poly[(R)-3-hydroxybutyrate]	0.003	10	3	1	0	0	0	0	0	0
[72]	Poly[(R)-3-hydroxybutyrate]	0.012	10	3	0	1	0	0	0	0	0
[72]	Poly[(R)-3-hydroxybutyrate]	0.00047	10	3	1	0	0	0	0	1	0
[72]	Poly[(R)-3-hydroxybutyrate]	0.0090	10	3	1	0	0	0	0	1	0
[72]	Poly[(R)-3-hydroxybutyrate]	0.018	10	3	0	1	0	0	0	1	0
[72]	Poly[(R)-3-hydroxybutyrate]	0.0010	10	3	0	1	0	0	0	1	1
[72]	Poly[(R)-3-hydroxybutyrate]	0.017	10	3	0	1	0	1	0	0	0
[44]	Premnaspirodiene	0.011	10	9	1	0	1	1	0	0	0
[44]	Premnaspirodiene	0.0090	10	9	1	0	1	1	0	0	0
[73]	Pyruvate	0.55	9	0	0	0	1	0	0	1	0
[46]	Pyruvate	0.0050	9	0	0	0	1	0	0	1	0
[74]	Reticuline	0.051	8(0)	16(3)	0	1	0	0	1	0	0
[75]	Ribitol	0.0020	5	2	0	0	1	0	0	0	0
[75]	Ribitol	0.027	5	2	1	0	1	0	0	0	0
[75]	Ribitol	0.017	5	2	1	0	1	0	0	0	0
[75]	Ribitol	0.021	5	2	1	0	1	0	0	0	0
[41]	Squalene	0.042	10	9	1	0	0	1	0	0	0
[76]	Taxadiene	7.7 × 10^-5^	12	8	0	1	0	1	0	0	0
[77]	Vanillin	0.0030	3	6	0	1	1	1	0	0	0
[75]	Xylitol	0.0070	5	2	1	0	1	0	0	0	0
[75]	Xylitol	0.014	5	2	1	0	1	0	0	0	0
[75]	Xylitol	0.014	5	2	1	0	1	0	0	0	0
[47]	Xylitol	0.27	5	2	0	1	0	0	0	0	1
[47]	Xylitol	0.29	5	2	0	1	1	0	0	0	1
[78]	β-carotene	4.5 × 10^-7^	10	14	1	0	0	0	0	0	0
[78]	β-carotene	2.9 × 10^-6^	10	14	0	1	0	0	0	0	0
[78]	β-carotene	0.00011	10	14	0	1	0	0	0	0	0
[78]	β-carotene	0.00036	10	14	0	1	0	0	0	0	0
[78]	β-carotene	0.0010	10	14	0	1	0	0	0	0	0

Note: Some papers show that the biosynthesis can be enhanced by supplementing additional precursors. In the parenthesis, we had listed the number of enzyme steps from the added intermediates to final products.

* Steps for ethylene was based on the arginine route.

## Result and Discussion

We constructed simple models which linked several numerical and ordinal variables that affected the yield of chemical production from *S. cerevisiae*. These ordinal variables consisted of the number of modified genes or pathways (OVE), the number of gene knockouts in known competitive pathways (KNO), nutrient source (NUT), intermediate (INT), cultivation mode (CUL), and oxygen availability (OXY). We described the yield of chemical production as the summation of these independent variables in Equation 2. We fitted Equation 2 and determined the coefficients of the variables using linear regression analysis of ~40 compounds. Although multiple data of production yields were often reported in each literature, the model only considered the best yield under a denoted experimental condition. Then, all experimental conditions were categorized by numerical and ordinal variables. The linear regression coefficients obtained for Equation 2 were given in Equation 4, such that:(4)

**Figure 2:**
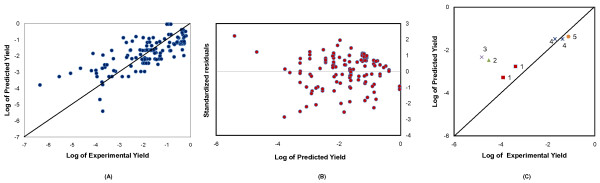
**Model results**. A) Plot of the actual logarithmic yields against the logarithmic yields generated by the regression model. The line drawn as diagonal to the plot is one-to-one and passes through the origin. The data points have an R^2 ^value of 0.55. B) Plot of residuals against fitted values. C) Model validation using newly published data (2010~2011) 1 - β-amyrin[22]; 2 - ascorbic acid [23]; 3 - monoterpene [24]; 4 - vanillin [25]; 5 - succinic acid [26].

The accuracy of obtained coefficients in Equation 4 was evaluated based on R^2 ^and the P-value. Here, we used a P-value of 0.1 as the limit below which the result was considered significant [14]. Out of the eight variables specified in our model, SEC, OVE, KNO, NUT, INT and CUL had P-value of less than 0.1. The summary of the P-value of each variable was listed in Table 3. Figure 2A showed a plot of the production yields obtained experimentally and those obtained from model prediction for the corresponding conditions. The correlation of this model to the dataset had an R^2 ^value of 0.55, which reflected the moderate discrepancy between reported yields and the model-predicted yields. Figure 2B plotted the residuals of model fitting. The residuals appeared to scatter around zero randomly, so the linear model was proper to describe the experimental data.

**Table 3:** Regression coefficients and P-values for *S. Cerevisiae *Model

	Model 1			Model 2			Model 3		
	With primary steps			Without primary steps			Ethanol as a primary metabolite		
Variable	Coefficient	P-value	**Std**. **Error**	Coefficient	P-value	**Std**. **Error**	Coefficient	P-value	**Std**. **Error**
**Intercept**	-1.53	0	0.42	-1.60	0	0.34	-1.73	0	0.41
**Primary step**	-0.01	0.76	0.04	-	-	-	0.003	0.93	0.03
**Secondary step**	-0.19	0	0.02	-0.19	0	0.02	-0.19	0	0.02
**OVE _C2_**	0.007	0.98	0.26	0.0003	0.99	0.25	0.05	0.84	0.24
**OVE _C3_**	0.52	0.07	0.29	0.50	0.079	0.28	0.56	0.05	0.28
**KNO _C2_**	0.31	0.08	0.18	0.31	0.078	0.18	0.37	0.03	0.17
**NUT _C2_**	0.73	0	0.18	0.73	0	0.18	0.71	0	0.17
**INT _C2_**	0.77	0.02	0.31	0.82	0.001	0.25	0.86	0.004	0.29
**CUL _C2_**	0.51	0.02	0.22	0.51	0.02	0.21	0.51	0.02	0.21
**OXY _C2_**	0.27	0.32	0.27	0.28	0.31	0.27	0.12	0.65	0.27

**Multiple R^2^**		0.55			0.55			0.58	

Interestingly, the number of enzymes in the primary pathway (PRI) did not significantly affect production yield (P-value = 0.76) (Table 3). This suggested that rate-limiting steps to increase chemical production flux often lay in the downstream pathway of central metabolism. The coefficient of SEC was negative. This suggested that the length of a pathway downstream of central metabolism negatively affected production yield. Specifically, addition of a new enzymatic step in a secondary metabolic pathway reduced product yield by 36% (for numerical variable SEC: ). A good demonstration of the effect of pathway length on product yield was found in the case of naringenin production [15]. With the following inputs of variables PRI = 10 (Galactose to PEP), SEC = 14 (i.e., 10 steps from PEP to phenylalanine; 4 steps from phenylalanine to flavanone), KNO = INT = CUL = OXY = category 1, NUT = Category 2; OVE = Category 3; the model calculated:

Yield = 10^-1.53- (0.01 × 10) + (-0.19 × 14) + 0.52+0.73 ^= 0.0009 (The reported experimental production yield was 0.00058). In most cases, our model-predicted yields were within the range of one order of magnitude compared to the experimental values.

Since the number of steps in central metabolism (PRI) did not significantly affect production yield, we computed another set of regression coefficients for Equation 2 without the variable PRI, to yield a simplified form Equation 5.(5)

As shown in Table 3, regression using Equation 2 with the exclusion of the variable PRI did not change the R^2 ^value. This result indicated that the number of enzymatic steps in primary metabolism did not significantly affect product yield. Presumably, fluxes in central metabolic pathways were typically high and robust [16], when compared to those downstream secondary pathways. It has been demonstrated recently that production of chemicals was significantly improved, only when the capacity of a downstream pathway was increased [17].

Metabolic engineering typically involves pathway modification [16-22] to shift metabolic fluxes into a desired product or to permit the use of an alternative carbon source. We defined the variable OVE, and KNO in Equation 2 to capture the effect of pathway overexpression, and deletion, respectively. The regression of experimental data using Equation 2 showed that the coefficients of OVE_C2 _and OVE_C3 _had positive values (Table 3). The model successfully captured the contribution of both pathway overexpression and gene deletions to increase product yield in *S. cerevisiae*. The high P-value of OVE_C2 _(0.98) indicated that statistically, the overexpression of a small number of genes (1-2) was uncertain to improve production yield. However, the coefficient of OVE_C3 _(= 0.52; P-value = 0.07) indicated the effectiveness of multiple gene modification to resolve the bottleneck steps. This observation is consistent to the fact that metabolic fluxes generally do not sensitively respond to changes of single enzyme activity, but are controlled by all key enzymes along the biosynthesis pathway. On the other hand, the regression coefficients of KNO_C2 _had positive value (= 0.31, P-value = 0.08), and thus the removal of competitive pathways could be effective to increase production yield.

It is a general knowledge that bioprocess conditions affect cellular viability and product yield. Our model suggested fermentation using a well-controlled bioreactor improved production yield by 3.2 times . The model further suggested that fermentation under anaerobic or microaerobic condition could enhance yield compared to aerobic fermentation. However, such enhancement was not statistically significant (P-value = 0.32). This observation could be explained by the fact that *S. cerevisiae *produced fermentative products (ethanol and glycerol) (Crabtree effect) [18,19] under aerobic and glucose-sufficient medium. Therefore, aerobic metabolism in *S. cerevisiae *could operate similarly to metabolism under oxygen-limited condition. The coefficient for the variable INT was 0.77, which represented that the supplementation of a precursor metabolite translated to an approximately six fold increase of the product yield (P-value = 0.02). Similarly, the addition of nutrients (such as yeast extract) also significantly increased production yield (the coefficient of NUT_C2 _was 0.73). The contributions of INT and NUT to product formation indicated that intermediates/nutrients provided building blocks or energy sources that reduced the rate-limiting steps in biosynthetic pathways.

We used Equation 2 to compute the production yield of chemicals according to the specifications listed in Table 2. We observed that, for ethanol production, the experimental values were generally higher than the empirical model predictions. In reality, the reported maximum ethanol yield could reach 0.5 mol C-ethanol/mol C-glucose [20], which could be several folds higher than model predictions. To mitigate this discrepancy, we re-categorized the ethanol synthesis pathway as the primary pathway to generate Equation 6.(6)

Regression of the data using Equation 6 improved the R^2 ^value from 0.55 to 0.58, demonstrating that ethanol could be better assumed as a central metabolite for *S. cerevisiae*. Using Equation 6, we predicted ethanol production based on a recent reference [21] by specifying PRI = 11, SEC = 1 (cellulose degradation step), OVE = C3, KNO = C1; NUT = C2, INT = C1, CUL = C1, and OXY = C2. The ethanol production yield calculated by Equation 6 was 0.31. This value was in good agreement with the reported values of ~0.4 [21].

### Model Applications and Limitations

The main application of the model is to predict the biosynthesis yield from *S. cerevisiae*. The model were validated by "unseen data" (Figure 2C) from some randomly selected new publications (2010~2011). The model predicted the yields based on the reported experimental conditions described by these papers [22-26]. Most yield data were close to model predictions. The predictive power of the model was consistent with the model quality described in Table 3.

**Figure 3:**
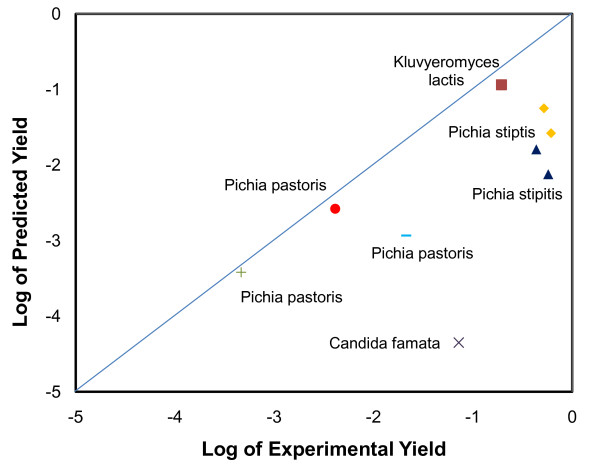
***S. cerevisiae *model prediction of biosynthesis yields for other industrial yeast species **[27-29,38-40]. Ethanol: ■ or ◆. L-lactic acid: ▲. Lycopene: ●. Riboflavin: **+ **or **×**. S-adenosyl**-**L**-**methionine: ─.

Furthermore, the model can reveal the metabolic features of *S. cerevisiae*. For example, the modified model Equation 6 showed that it was better to treat ethanol pathway as the primary routes in cell metabolism, because of the strong ability for ethanol fermentation by yeast, possibly due to long-term process for selecting yeast as alcohol producer through human history. The model can also be useful for comparing the productivity among other yeast species (Figure 3). For example, riboflavin producer, *Candida famata*, exhibits a high riboflavin productivity (2~3 order of magnitude higher than model prediction) [27]. *Pichia pastoris*, a common species for protein expression, shows high S-adenosyl-L-methionine productivity if a large amount of the intermediate methionine was repeatedly added in the medium [28]. Besides, *Pichia stipitis *also has high yields of L-lactic acid and ethanol from glucose and xylose [29]. Figure 3 demonstrated that some yeast species were able to explore their native pathways for biosynthesis of certain products with extraordinary efficiency (better than *S. cerevisiae*), therefore, these yeast species may be alternative hosts for certain biotechnology applications.

The accuracy of the model predictions for some products could be poor due to several limitations during model development. First, the category was a rough estimation of experimental conditions especially for variables related to gene modifications (OVE and KNO), and the yields could be very different even in the same category. Second, some products, despite large synthesis rates, were either not very stable or difficult to accumulate in a large quantity due to consumptions by downstream pathways or product degradations (e.g., Glycerol 3-phosphate [30]). Their yields could be significantly lower than model predictions even though the actual flux to the product was high. Third, the coefficient β_SEC _from model regression could not account for the big variances of biosynthesis efficiency or potentially feedback inhibitions in secondary pathways. For example, butanol synthesis is significantly improved via non-fermentative amino acid pathways compared to traditional acetyl-CoA routes [31], because amino acid synthesis pathways in microorganisms are more effective than other heterogeneous pathways. Fourth, because of limited information from the references, the yield calculation could not precisely include the CO_2 _fixation (e.g., overexpression of the native carboxylase pathway: pyruvate + CO_2 _→ oxaloacetate) [32] or the nutrients utilization in the rich medium. Fifth, the model neglected enzyme steps related to energy metabolism (such as ATP and NADPH synthesis), while cofactor imbalance can also affect the product yields.

### Comparison to the previously published *E. coli *model [33]

Recently, we have constructed the *E. coli *model using same modeling approach. Compared to the *E. coli *model, *S. cerevisiae *shows several differences: 1. Oxygen conditions made a more significant impact on biosynthesis yield in *E. coli *than that in *S. cerevisiae*; 2. The genetic modification in *E. coli *had higher uncertainty for metabolic outcomes; 3. For metabolic pathways from precursors to final products, loss of yield per biosynthesis step (~30%) in *S. cerevisiae *is higher than that in *E. coli *(10~20%). Interestingly, *E. coli *model states that primary metabolism influences product yield (a relatively small P-value of 0.06) which indicates the balance of precursor production from central metabolism is also an important consideration for metabolic engineering of *E. coli*. For example, it has been demonstrated that lycopene production with *E. coli *was enhanced by redirecting the carbon flux from pyruvate to G3P [34], but feeding other central metabolite precursors (such as pyruvate) could not improve lycopene production. On the other hand, the *S. cerevisiae *model indicates that it is less likely that the number of steps in central metabolism play a bottleneck role in the production of metabolites derived from it, while the bottlenecks are more likely in the secondary pathways (from central precursors to the final product). Therefore, the metabolic strategies should focus on the secondary pathways to have a better chance for increasing final yield. Although modification of central metabolism may affect microbial physiologies, a few studies indicate the robustness of the central metabolism in *S. cerevisiae *because of its importance to cell vitality. For example, *S. cerevisiae *may maintain central metabolic fluxes via gene duplication and alternative pathways under different environmental and physiological conditions [16,35]. Therefore, the inflexibility of central pathways in *S. cerevisiae *is likely to render metabolic engineering strategies ineffective when targeting enzymes in central metabolism. In general, the unique metabolic features of yeast and bacteria can be of important consideration when choosing a production host.

## Conclusions

Although *S. cerevisiae *has been widely used as a robust industrial organism for metabolic engineering applications, many metabolic features of this organism for biosynthesis under various conditions remain unknown. In this study, the statistic model for yeast biosynthesis permits *a priori *calculation of the final product yield achievable by current biotechnology. Unlike other *in silico *models based on mass balance or thermodynamics (such as FBA model) [36,37], our model is based on a statistical analysis of published data using numerical and ordinal variables (categorized experimental conditions). The model has three applications. 1. The yield prediction takes into account the genetic design of the microbial host system and the "suboptimal" conditions under which the fermentation process occurs. 2. The model may identify effective metabolic strategies and at the same time, quantitatively provide the degree of uncertainty (i.e., possibility for failure). For example, statistical analysis shows that, for *S. cerevisiae*, metabolic bottlenecks may be more likely to be in the secondary metabolic pathways rather than primary pathways, and thus it can narrow down the genetic targets and avoid futile work. 3. This model may be used to qualitatively benchmark yields of different engineered production platforms.

## Competing interests

The authors declare that they have no competing interests.

## Authors' contributions

Conceived and designed the models: YJT, AMV, and EL. Data collection and analysis: AMV and YX. Wrote the paper: AMV, YJT and EL. All authors have read and approved the final manuscript.
